# The emergence of sarcoptic mange in Australian wildlife: an unresolved debate

**DOI:** 10.1186/s13071-016-1578-2

**Published:** 2016-06-02

**Authors:** Tamieka A. Fraser, Michael Charleston, Alynn Martin, Adam Polkinghorne, Scott Carver

**Affiliations:** School of Biological Sciences, University of Tasmania, Sandy Bay, 7001 TAS Australia; Centre for Animal Health Innovation, Faculty of Science, Health, Education and Engineering, University of the Sunshine Coast, 91 Sippy Downs Drive, Sippy Downs, 4556 QLD Australia; School of Information Technologies, University of Sydney, Camperdown, 2006 Sydney Australia

**Keywords:** *Sarcoptes scabiei*, Wombat, Network, Phylogeny, One Health, Conservation Medicine

## Abstract

Due to its suspected increase in host range and subsequent global diversification, *Sarcoptes scabiei* has important implications at a global scale for wildlife conservation and animal and human health. The introduction of this pathogen into new locations and hosts has been shown to produce high morbidity and mortality, a situation observed recently in Australian and North American wildlife.

Of the seven native animal species in Australia known to be infested by *S. scabiei*, the bare-nosed wombat (*Vombatus ursinus*) suffers the greatest with significant population declines having been observed in New South Wales and Tasmania. The origins of sarcoptic mange in Australian native animals are poorly understood, with the most consistent conclusion being that mange was introduced by settlers and their dogs and subsequently becoming a major burden to native wildlife. Four studies exist addressing the origins of mange in Australia, but all Australian *S. scabiei* samples derive from only two of these studies. This review highlights this paucity of phylogenetic knowledge of *S. scabiei* within Australia, and suggests further research is needed to confidently determine the origin, or multiple origins, of this parasite.

At the global scale, numerous genetic studies have attempted to reveal how the host species and host geographic location influence *S. scabiei* phylogenetics. This review includes an analysis of the global literature, revealing that inconsistent use of gene loci across studies significantly influences phylogenetic inference. Furthermore, by performing a contemporary analytical approach on existing data, it is apparent that (i) new *S. scabiei* samples, (ii) appropriate gene loci targets, and (iii) advanced phylogenetic approaches are necessary to more confidently comprehend the origins of mange in Australia. Advancing this field of research will aid in understanding the mechanisms of spillover for mange and other parasites globally.

**Electronic supplementary material:**

The online version of this article (doi:10.1186/s13071-016-1578-2) contains supplementary material, which is available to authorized users.

## Background

The spread of pathogens from endemic to novel host foci, otherwise known as spillover, is one of the most significant threats to the health of both animals and humans, globally [1, 2]. Approximately 61 % of known human pathogens are zoonotic and up to 90 % of pathogens infecting animals are transferable between other animal species [3]. Identification of spillover reservoirs is important for management attempts to intervene in further pathogen pollution and determining if long established diseases are invasive. Indeed the latter of these can be critical for justifying management, particularly for establishing whether a pathogen is “native” or invasive to a host or region and if it warrants control in wildlife. Modern molecular techniques, including phylogenetic comparisons and metagenomics, have revolutionised our ability to identify spillover and characterise pathogens [4, 5].

In this review, we focus on an important example of disentangling the origins of a pathogen causing significant disease burden. Sarcoptic mange (causative agent *Sarcoptes scabiei*) is a major disease of Australian wildlife, particularly to wombats (bare-nosed/common and southern hairy-nosed), and also impacts humans, domestic animals, and other Australian wildlife, with negative economic outcomes [6]. *Sarcoptes scabiei* is a parasitic astigmatid ectoparasite which feeds off skin cells and serum as it burrows into the epidermal and dermal layer of its host. It has an extremely wide host range, infecting over 104 mammal species, and is a global contributor to the world’s burden of parasitic infestations [7]. Similar to what has been seen for emerging viral diseases, this mite has had an important role in shaping host populations, causing the collapse of several host species around the world [8]. *Sarcoptes scabiei* is known to infest both humans and animals; in the former, the resulting disease is referred to as scabies, whereas infestation of domesticated animals and wildlife is referred to as mange [9]. The broad host range of sarcoptic mange commonly includes domestic dogs, livestock (e.g. cattle, pigs, goats, camelids) and wildlife (e.g. red foxes, coyotes, wolves, deer, bobcats, wombats, koalas and wallabies) and poses an important welfare and economic burden, globally [6, 10]. More recently the need for greater research on this largely neglected pathogen has been highlighted owing to its resurgence and emergence in several areas across the globe [11, 12], leading to its classification as a wildlife emerging infectious disease [2], particularly owing to host range expansion in Australia and North America.

The origins and even endemicity of this pathogen have been the source of much debate. Here, we review the genetic evidence of host specificity and cross-species transmission of *S. scabiei*, examine the strengths and limitations of the existing literature around this topic, and propose critical directions for more clear and concise answers into the degree of variation (information) provided by genetic data. We focus on Australia as a case study, owing to the importance of this pathogen at a national scale, particularly in wildlife conservation and human and domestic animal health. However, the broader principles apply to mange in many other regions globally, (e.g. North America) and other pathogens with debated origins (e.g. *Chlamydia* in koalas).

## Historical origins and epidemiology of mange in Australian wildlife

Questions over the origin, reservoirs and transmission of *S. scabiei* mites in and between Australian wildlife host species have been ongoing for nearly two centuries [13]. These questions have persisted owing to their perceived importance for detaining the reservoirs of infections and controlling this pathogen. General perceptions have been that mange was introduced into Australia by European settlers and/or their domestic dogs [13]. Mange is known to affect a number of Australian wildlife species including the koala [14], agile wallaby [15], swamp wallaby [16], southern brown bandicoot [17], dingo [18, 19] and the bare-nosed and southern hairy-nosed wombat [13]. The earliest records of mange on an Australian animal date back to Latreille (1818), where mites infecting a wombat held at the *Muséum national d’Histoire naturelle* in Paris, were identified as identical to *S. scabiei* found on a human male, however it is possible that mange was contracted in translocation [13]. It was not until 1937 that mange was first identified in a New South Wales bare-nosed wombat population which had undergone a large population decline, most likely due to the disease [20].

There has been considerable debate and anecdotal evidence surrounding the role that foxes and wild dogs may have in the transmission of mange to Australian wildlife [21]. The red fox was introduced into Australia in 1850 and is known to be a host to *S. scabiei* [18]. Since (i) mites are capable of surviving in low temperatures and high relative humidity for extended periods of time of potentially up to three weeks [22, 23], and (ii) it has been documented that canids periodically enter wombat burrows, it is possible that the route for transmission between both canids and wombats occurs via burrows [13]. Furthermore, domestic dogs have been shown to contract mange after predating upon mangy wombats [20]. Some suggestions have been made that canids may be necessary for disease persistence in marsupials [24, 25]. In contrast to these hypotheses, persistent disease is observed in Tasmanian bare-nosed wombats where foxes are considered absent [26]. Thus, evidence suggests mange can persist in Australian wombats, and possibly other wildlife, with or without the involvement of canids. *Sarcoptes scabiei* infestations have also been widely reported in Australian indigenous communities, domestic dogs and livestock, with ongoing economic costs associated with human health and animal health and production [6, 27]. Extensive studies of this ectoparasite in humans, pigs and dogs have recently been performed with an emphasis on developing a vaccine [6, 28–31].

## Prevalence and pathology of sarcoptic mange in Australian wildlife

The pathology associated with mange in Australian marsupials is consistent with other animals globally, suggesting the symptomology is not unique to Australia (and by extension the mite, or strain of mite, is also not distinct). Symptoms include irritation, inflammation, hyperkeratosis, alopecia, pruritis, dermatitis and lesions that are typically coupled with pneumonia and secondary infections [7, 32]. Although the koala, wallaby, possum, bandicoot and wombat all have shown signs of mange, the most severe pathology and consequent conservation threat is to the bare-nosed and southern hairy nosed wombats, as mange has been shown to cause localised extinction in isolated populations [25]. Of the three species of wombat living in Australia, the bare-nosed wombat appears to be more susceptible, suffering higher morbidity and mortality [25]. For example, mass declines of 70 % in bare-nosed wombat populations in New South Wales [20] and > 80 % in a bare-nosed population in Tasmania [33] (Martin et al., in prep), have been documented. It is likely that numerous other population declines have occurred but gone undocumented owing to the absence of reliable information on the prevalence and distribution of individual wombat populations. However, it is also notable that other populations of bare-nosed wombats may experience more variable impacts, such as endemic dynamics with low background mortality. The severity of this disease impacts on wombats, and the ability of this pathogen to drive catastrophic declines, have contributed to the classification of this pathogen to likely be an introduced pathogen to wombats, and Australian wildlife more broadly, and have also spurred periodic genetic studies to address this.

## Genetic attempts to identify the origins of mange in Australian wildlife

To date, attempts to answer questions concerning the origin of mange in Australian wildlife have largely centred on the use of individual genetic markers to identify similar, if not genetically identical, mites between wildlife and humans in Europe, Asia and Australia. Skerratt et al. [34] identified mites from wombats, dogs and humans in Australia to have similar 12S rRNA gene sequences and concluded that European settlers and their domestic dogs introduced mange into Australian wildlife. Following this study, Walton et al. [35] expanded not only the known host range of mange in Australia but targeted three different gene regions of *S. scabiei* for genetic comparisons: cytochrome *c* oxidase subunit I (COX1), 16S rRNA gene and microsatellites. Analysing microsatellites and COX1 sequences, Walton et al. [35] revealed that wombat *S. scabiei* sequences separated into their own subclade within a human and animal clade (dog, human, chimp, wallaby, wombat and fox). However, conflicting results occurred for the wombat sample when evaluating the 16S rRNA gene sequences, as the mite extracted from the wombat was identical to a canine *S. scabiei* 16S rRNA gene sequence [35]. More recently two studies [36, 37] based on data obtained from *S. scabiei* from France concluded that *S. scabiei* was introduced into Australia by European settlers based on a single French human *S. scabiei* sequence being identical to the reference *S. scabiei* var *wombatii* by 12S rRNA gene, and clustering of French and Australian human mites based upon COX1 sequences. An obvious limiting factor in these studies is the lack of new Australian samples used to accurately confirm their conclusions, as the 12S rRNA gene and COX1 sequences used were originally obtained by Skerratt et al. [34] and Walton et al. [35].

Of these four Australian marsupial studies [34–37], spanning nearly 15 years, it is notable that their conclusions have been strongly influenced by the choice of molecular marker gene and the geographical locations of both animal and human mite samples. Two of these studies [34, 37] have used the 12S rRNA gene, but is it increasingly recognised that this locus is relatively uninformative of phylogenetic structure among host species and populations for *S. scabiei* [35, 38, 39]. Contrastingly, gene loci COX1, 16S rRNA and microsatellites, used by Walton et al. [35] had greater genetic discrimination and, accordingly, the authors identified significant genetic structure based upon host species and geographical location. Additionally, the COX1 gene contains numerous sites where single nucleotide polymorphisms can occur in this relatively conserved part of the mitochondrial genome, with a mutation rate rapid enough to distinguish between closely related species [35, 40]. Although microsatellites have the potential to support investigations of genetic structure of natural populations where environmental barriers, mating systems and historical processes can alter the genetic structure [41], Walton et al. [35] clearly revealed higher genetic discrimination using COX1 compared to their microsatellite results. Furthermore Walton et al. [35] was able to demonstrate significant relationships between their 16S rRNA gene haplotypes, similar to COX1, which is interesting since the use of 16S rRNA is similar to 12S rRNA, as it is valuable for the identification of species but limited for intra-species analysis [42].

Beyond the choice of gene markers for studying the genetic relationships of mites from different hosts, the simple fact is that adequate sampling is still a major limitation to answering questions of this nature. Collectively, the mites used for these Australain *S. scabiei* sequence comparison studies include a total of eight wombats from Victoria, one wombat from South Australia, 17 humans and ten dogs from the Northern Territory, along with samples from outside of Australia, including: ten humans from Panama, ten dogs from the USA, one chimpanzee from Tanzania, one fox from Sweden, and two dogs, one pig and 83 humans from France [34–37]. An expansion of sampling and molecular typing, particularly from a range of Australian marsupials and geographically distinct wombat populations, is clearly required for convincing phylogenetic inference.

## Global attempts to study *S. scabiei* origin and spillover

More broadly there have been three approaches either to understand spillover or to infer origins of *S. scabiei* in the global literature: mite morphology, experimental cross-infections, and genetics. Minor morphological differences have classified *S. scabiei* into varieties (pathovars) [6, 23, 36, 43] with the presence or absence of dorsal and ventrolateral spines used as the primary differentiator [23]. These pathovars are simply named: *S. scabiei* var *hominis*, *S. scabiei* var *canis* and *S. scabiei* var *animal*, which can be distinguished further depending on the specific animal infested (e.g. *S. scabiei* var *wombatii*). Cross-infestations of mites between different host species have also been shown to occur using this identification of pathovars, however, these documented spillover events have typically been self-limiting [10, 29, 44–46]. In terms of phylogenetic informativeness, a range of different gene loci have been used to attempt to answer questions about the relationship between mites isolated from different hosts. Outside Australia, genetic studies using a range of different genetic markers have revealed conflicting conclusions over whether geographic location and host has an impact on *S. scabiei* genetic structure [32, 38, 47, 48]. A detailed comparison of different gene loci can be seen in Table 1, with a total of 17 studies occurring during a 16 year period, spanning across 19 countries and 34 animal species. All studies were attempting to answer whether mites were genetically different depending on the host they were infecting and/or whether biogeographical separation existed. General conclusions from all gene targets include that (i) microsatellites identify distinctive host separation [35, 47, 49–52], (ii) COX1 and 16S are consistent with host and location separation, with human specific mites indicating higher species separation based on location according to COX1 [32, 35, 36, 42, 53, 54], (iii) ITS-2 and 12S should only be used for *S. scabiei* identification and that a single species of mite infects all animals and humans [32, 34, 37, 38, 42, 53, 55, 56], and (iv) genes encoding for glutathione S-transferase-1 and voltage-sensitive sodium channels (GST1 and VSSC, respectively) might be a good indicator for host-related variation and resistance [57]. Interestingly, Erster et al. [57] found that COX1 did not play a role in mite host-specific separation in this particular study, which is contrast to other COX1 studies [32]. With genetic studies mentioned in Table 1 producing variable results, there is a clear need for increased consensus in the literature on the choice of genetic loci to address questions of *S. scabiei* spillover and identify origins.

**Table 1:** Publicly available studies that have attempted to identify if *S. scabiei* can be genetically separated based upon location and/or host

Study	Host (Location)	Gene Target/Conclusions
Zahler et al. (1999) [55]	*Bos taurus* (Germany) *Camelus dromedarius* (Germany) *Canis lupus familiaris* (USA, India, Malaysia, New Zealand) *Lynx pardinus* (Sweden) *Nyctereutes procyonoides* (Japan) *Rupicapra rupicapra* (Austria) *Sus scrofa* (Germany, Belgium, Spain) Vombatidae (Australia) *Vulpes vulpes* (Sweden, Germany)	ITS-2: No separation due to location or host
Walton et al. (1999) [47]	*Canis lupus familiaris* (Australia, USA) *Homo sapiens* (Australia, Panama) *Vombatus ursinus* (Australia)	Microsatellites: Human and dog derived mites cluster by host rather than location.
Skerratt et al. (2002) [34]	*Canis lupus familiaris* (Australia) *Homo sapiens* (Australia) *Vombatus ursinus* (Australia)	12S rRNA: Wombats, dogs and humans had similar sequences.
Berrilli et al. (2002) [53]	*Rupicapra pyrenaica* (Spain) *Rupicapra rupicapra* (Italy) *Vulpes vulpes* (Italy, Spain)	ITS-2: No host or geographical separation. 16S rRNA: Indicated significant differences between locations.
Walton et al. (2004) [35]	*Homo sapiens* (Australia, Panama) *Canis lupus familiaris* (Australia, USA) *Macropus* (Australia) *Pan troglodytes* (Tanzania) *Vombatus ursinus* (Australia) *Vulpes vulpes* (Sweden)	16S: Produced three groups: (i) human mites from Panama; (ii) human mites from Australia; (iii) mixed human and animal mites. COX1: Produced three groups: (i) human mites from Panama; (ii) human mites from Australia; (iii) mixed human and animal mites. Microsatellites: Separated human mites into two distinct geographical clusters and further divided the animal mites into hosts groups.
Soglia et al. (2007) [49]	*Capra ibex* (Italy) *Cervus elaphus* (Italy) *Martes foina* (Italy) *Martes martes* (Italy) *Ovis gmelini* (Italy) *Rupicapra pyrenaica* (Spain) *Rupicapra rupicapra* (Italy) *Sus scrofa* (France) *Vulpes vulpes* (Italy, Spain)	Microsatellites: Low levels of cross infections. Not strongly supportive of geographical separation within same host-specific varieties.
Gu & Yang (2008) [56]	*Oryctolagus* (China) *Sus scrofa* (China)	ITS-2: Single heterogeneous species.
Alasaad et al. (2009) [38]	*Capra ibex* (Italy) *Capra pyrenaica* (Spain) *Cervus elaphus* (Italy) *Martes foina* (Italy) *Ovis aries musimon* (Italy) *Rupicapra pyrenaica* (Spain) *Rupicapra rupicapra* (Italy) *Sus scrofa* (Italy, France) *Vulpes vulpes* (Italy, Spain, Switzerland)	ITS-2: Not suitable to identify genetic diversity among mites from different animals in different locations: monospecific.
Rasero et al. (2010) [50]	*Capra ibex* (Italy) *Capra pyrenaica* (Spain) *Cervus elaphus* (Italy) *Martes foina* (Italy, Spain) *Martes martes* (Italy) *Ovis aries musimon* (Italy) *Ovis aries musimon* (Italy) *Rupicapra pyrenaica* (Spain) *Rupicapra rupicapra* (Italy) *Sus scrofa* (Italy, France) *Vulpes vulpes* (Italy, Spain)	Microsatellites: Mites clustered into herbivore, carnivore and omnivore derived mite populations and the level of genetic exchange between mites from different locations is related to geographical distance.
Gakuya et al. (2011) [52]	*Acinonyx jubatus* (Kenya) *Connochaetes taurinus* (Kenya) *Eudorcas thompsonii* (Kenya) *Panthera leo* (Kenya)	Microsatellites: Host-taxon specification with potentially predator/prey association.
Alasaad et al. (2011) [44]	*Capreolus capreolus* (Spain) *Cervus elaphus* (Spain) *Rupicapra pyrenaica* (Spain) *Vulpes vulpes* (Spain)	Microsatellites: Herbivore, carnivore and omnivore separation.
Amer et al. (2014) [32]	*Bos taurus* (Egypt) *Bubalus bubalis* (Egypt) *Oryctolagus* (Egypt) *Ovis aries* (Egypt)	ITS-2: No host segregation COX1: Host adaptation and geographically separated mites 16S: Shows host adaptation and geographically separated mites.
Zhao et al. (2015) [42]	*Canis lupus familiaris* (China) *Homo sapiens* (China)	16S: Differentiate *S. hominis* from *S. animal* populations, but not as effective as COX1. COX1: Classified mites by different hosts with *S. hominis* further divided based on locations. ITS-2: No host or geographical preference.
Makouloutou et al. (2015) [54]	*Canis lupus familiaris* (Japan) *Capricornis crispus* (Japan) *Martes melampus *(Japan) *Meles anakuma* (Japan) *Nyctereutes procyonoides viverrinus* (Japan) *Procyon lotor* (Japan) *Sus scrofa leucomystax* (Japan)	ITS-2: Only good for identification of causative agent. 16S: Showed minor genetic differences regardless of hosts in Japan. COX 1: Showed minor genetic differences regardless of hosts in Japan.
Erster et al. (2015) [57]	*Canis aureus* (Israel) *Erinaceus concolor* (Israel) *Orictolagus cuniculus* (Israel) *Vulpes vulpes* (Israel)	COX1: Did not indicate host preference. GST1: Differences in host preference. VCCS: Differences in host preference.
Andriantsoanirina et al. (2015) [36]	*Homo sapiens* (France) *Canis lupus familiaris* (France)	12S rRNA: Using Skerratt et al. [34] haplotypes concluded that a single French human mite sequence was identical to the reference *S. scabiei* var *wombatii*.
Andriantsoanirina et al. (2015) [37]	*Homo sapiens* (France)	COX1: Identified three genetically distinct clades: two clades exclusive to humans and one clade with a mix of both animals and humans. One of the two human mite clades had a mix of Australian and French samples.

## Reanalysis of Australian studies

In addition to improvements of choice of genetic loci, analysis by contemporary analytical approaches can also contribute value to the emerging picture of mange origins in Australian wildlife. While it is beyond the scope of this review to add new genetic data, we apply a contemporary analytical approach to existing data. In order to represent the, often conflicting, phylogenetic signal in the available data, we turn to phylogenetic networks. In such networks, groups of taxa are split by sets of parallel lines, whose lengths correspond to the strength of phylogenetic signal splitting the taxa in that way, rather than simply by single branches of a tree [58]. By including bootstrap values greater than 80, the robustness of each network split can be analysed.

**Fig. 1:**
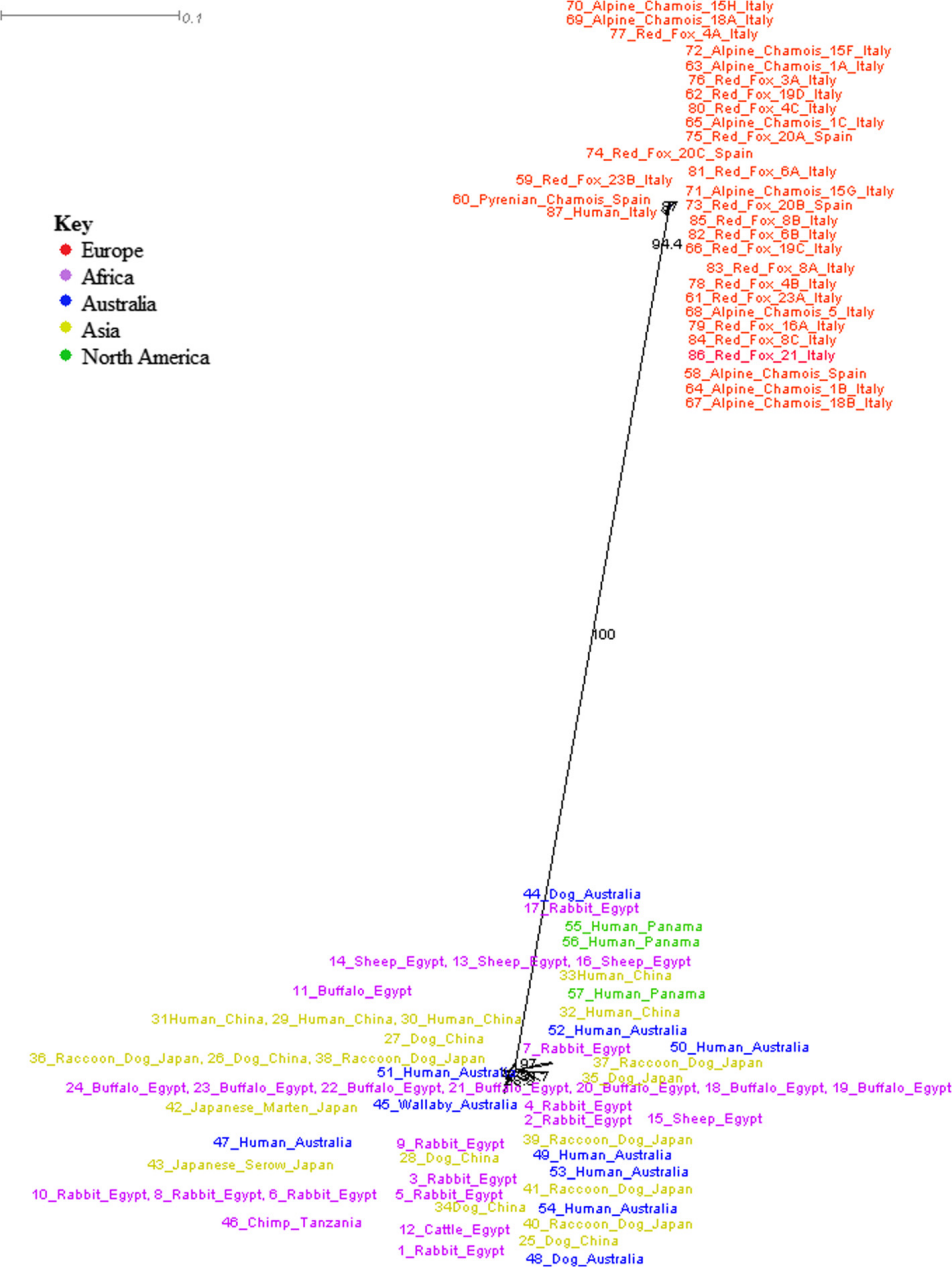
Neighbour-net analysis using SplitsTree of publicly available 16S rRNA gene sequences retrieved from GenBank (July 2015). Bootstrap values greater than 80 are included. Human and animal *S. scabiei* mites from Europe cluster away from other global *S. scabiei* mites. The Australian derived mites are shown to cluster closely with Asian and African mites, which conflicts with the assumption that Australian mites are consequential to European origins. There is limited network support for internal subclades in both the European clade and the Asian, Australian, African and North American clade

**Fig. 2:**
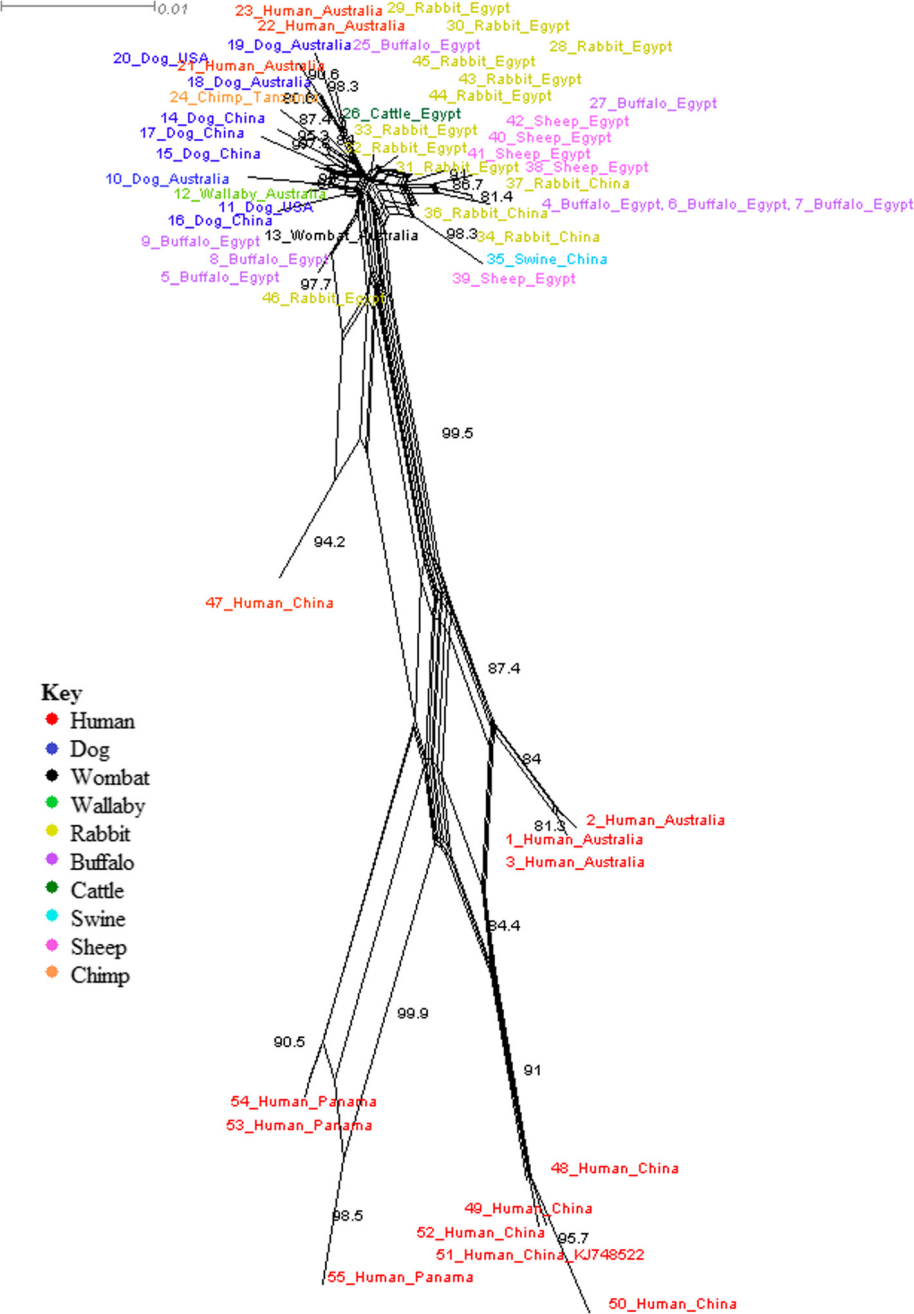
Neighbour-net analysis using SplitsTree of publically available COX1 sequences retrieved from GenBank. Bootstrap values greater than 80 are included. COX1 sequences analysis supports host-separation rather than geographic location is the biggest influence on *S. scabiei* diversity. Dog, wallaby and wombat sequences are shown to be clustering closely. The majority of sequences are branching away independently within both clades as unique sequences

To understand how Australian mites cluster in the global mite population using a phylogenetic network, *S. scabiei* 16S rDNA and COX1 were obtained from GenBank for neighbour-net tree analysis using SplitsTree (version 4.13.1) [58]. Sequences from human and animal *S. scabiei* mites were obtained across Italy, Spain, China, Egypt, Australia, Panama, Japan, North America and Tanzania (Additional file 1). The outcomes from these two networks targeting these two genetic loci produce slightly different results. 16S rRNA gene sequence analysis showed two very distinct clades; sequences from human and animal European *S. scabiei* in one clade and sequences from human and animal *S. scabiei* from the rest of the world in the other clade (Fig. 1). Within the non-European clade there is limited support for further supplementary subclades. Interestingly, *S. scabiei* mites from Australian hosts are shown to be clustering very closely to Egypt, Japan and China derived mites. This may suggest that the once thought European origin of Australian *S. scabiei* could be incorrect, and perhaps the Australian *S. scabiei* associates more with Asian roots, which are clearly separate from the European derived mites. COX1 sequence analysis did not produce the same location separation as did the 16S rRNA gene analysis, but rather showed separation of human and animal sequences, except for three human *S. scabiei* mites which were found in the animal clade (Fig. 2). Due to the lack of European derived mites for COX1, it is hard to accurately conclude whether European origins have an influence on this clade separation. Comparing the animal host clade of COX1 sequence analysis to the non-European clade of 16S rRNA gene sequence analysis, COX1 produced higher internal clade support for sequence separation than 16S rDNA. This suggests that COX1 may be more selective for higher divergence of *S. scabiei* than 16S rRNA gene.

Both neighbour-nets do produce an overall interpretation that geographic location and host species play a distinct role in mite separation. This supports that *S. scabiei* is frequently host-specific with periodic host spillover events. An interesting feature to note is that one study that used human *S. scabiei* mites from China (sample numbers 47–52) analysed only 45 % (approximately 317 bp) of the COX1 sequence compared to the longer sequence (approximately 1,448 bp) analysed by others (sample numbers 15–17 and 34–37). The remaining sequences were all roughly around 747 bp. Trimming all available COX1 sequences to 314 bp for SplitsTree analysis did not produce any significant changes to the overall outcome. However, the wallaby and wombat *S. scabiei* sequences were not separated in the neighbour-net as unique single sequences, but rather clustered on a node within the network. This emphasises the additional value of sequencing larger gene sequence fragments where possible, since phylogenetically informative areas of the gene may be excluded when limiting sequence length, which in turn may strongly influence the outcomes of such analysis.

**Fig. 3:**
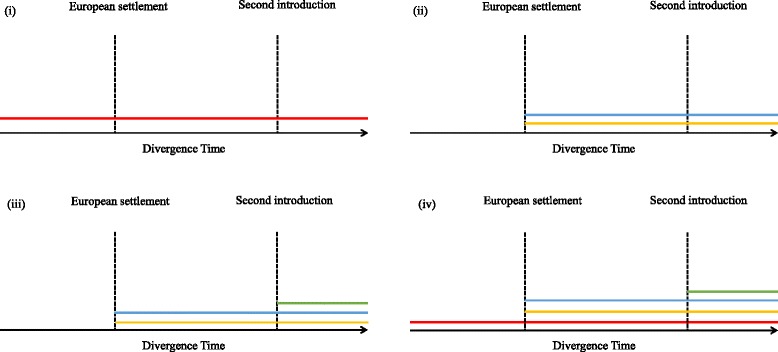
Representation of four different scenarios of how mites were introduced into Australian wildlife. Each line represents a different host, introduction period and are genetically unique. (i) *S. scabiei* was already present in Australian wildlife via the dingo prior to European settlement (ii) a single manifestation from European settlers and their domestic dogs (iii) after initial European settlement, a second and new introduction of *S. scabiei* was introduced from other regions across the world and (iv) combinations of all three situations (illustrating all three at once). Colours indicate species as follows: *red* - dingo, *yellow* - European domestic dogs, *blue* - European settlers, and *green* - second introduction of mites from other ethnic countries. Each of these possible scenarios would produce different clade structures on a phylogenetic tree, respectively as follows: (i) a single Australian *S. scabiei* subclade within the larger *S. scabiei* phylogeny with divergence time pre-dating European arrival, (ii) single or two Australian *S. scabiei* subclades within the larger phylogeny with divergence time associated to European arrival, (iii) a further subclade within (ii) associated to reintroduction times, and (iv) a single *S. scabiei* subclade within the larger *S. scabiei* phylogeny distinctly separate from the smaller subclades associated to European settlement and other more recent introductions

By adding additional new *S. scabiei* samples and solving some of the genetic loci problems, as discussed, greater consensus may be reached as to the origin of mange in Australian wildlife. We propose that there are several alternative hypotheses that may be revealed about the mechanism of spillover, its frequency, and timescale from improved *S. scabiei* phylogenetics: (i) mange was already present within Australian wildlife such as dingos before the arrival of European settlers; (ii) there was a single introduction event from original European settlement; (iii) there were multiple events of introduction since European settlement from other ethnic regions; and (iv) combinations of these hypotheses (Fig. 3, illustrating the combination of all three). To resolve these hypotheses, supplementary sampling needs to occur, including increased sampling from dingos, other canids and wombats to answer questions over the genetic diversity of *S. scabiei* mites within Australian wildlife. Additionally, sampling from humans, canids and other animals that are prone to high morbidity of *S. scabiei* in endemic countries would help resolve the genetic timeline globally.

Alternatively, the advent of whole genome sequencing of nuclear or mitochondrial DNA may replace these gene-specific analyses altogether [59, 60]. In the absence of whole genome sequencing, we recommend using COX1 gene molecular typing for *S. scabiei* host species specific separation. Additionally, perhaps rather than simply asking “are the mites different?” more explicit hypothesises about likely mechanisms, timescales and frequency of spillover or origins should be addressed. For example, it is notable that while the past studies have either agreed or disagreed on host and/or location to be a definitive key to different *S. scabiei* mites, the use of sequence data to infer divergence times using ancestral state reconstructions has never been addressed [61].

## Conclusions and future directions

New pathogens in a novel organism have the potential to cause high morbidity and mortality to animals that have not previously been exposed or have evolved defences [62]. Such knowledge can also be critical for justifying disease management, owing to perceived invasiveness. The settlement of Europeans and their livestock into Australia since 1788 has introduced new pathogens, with sarcoptic mange in Australian wildlife proposed to be one of several important examples [13]. This scenario is unlikely to be unique in Australia with growing molecular evidence that the obligate intracellular bacteria, *Chlamydia pecorum*, and major pathogen of the iconic Australian marsupial, the koala, may have origins in spillover from introduced livestock carrying this pathogen [63, 64].

To date, the most convincing conclusion is that sarcoptic mange in Australia was introduced by settlers and their dogs, and subsequently became a major disease burden to native wildlife. This review has discussed the conflicting results of phylogenetic studies of sarcoptic mange and highlighted the need to establish a more consistent and robust set of genomic loci for analysis. We conclude that of all available gene loci that have been used, a combination of both genomic (e.g. microsatellites) and mitochondrial (COX1) loci should be combined for host and location separation to have the best chance to eliminate phylogenetic conflict. Genes encoding for GST1 and VSSC may be equally important as these genes are related to immune resistance; however, further research is needed to confirm this, and to expand available sequences for comparison. In light of this reanalysis, perhaps an additional question to explore is “since genetic differences exist between mites infecting different hosts and locations, do these differences occur in key genes that can influence disease states and pathogenicity, and is there a more selective gene that can better identify mite variation?”. This can also be simplified as simply that better markers are needed, along with the right samples to assess them.

Mange in Australian wildlife illustrates the importance of sarcoptic mange due to its continual increase in host range and global diversification [2]. Future genetic and phylogenetic research will contribute valuable knowledge applicable to wildlife conservation and the health to both humans and animals infected with *S. scabiei* (a Conservation Medicine and One Health framework).

## Additional file

Additional file 1: 16S rRNA gene and COX1 sequences retrieved from GenBank. Each sequence is labelled as follows: Representative number associated to its corresponding Neighbour-net tree_Host_Location_Accession Number (DOC 85 kb)

## Abbreviations

12S rRNA: 12S ribosomal RNA
16S rRNA: 16S ribosomal RNA
GST1: glutathione S-transferase-1
VSSC: voltage-sensitive sodium channel
COX1: cytochrome *c* oxidase subunit 1
USA: United States of America
