# Supplementary material for: The emergence of sarcoptic mange in Australian wildlife: an unresolved debate
Source: Parasit Vectors. 2016 Jun 2;9:316. doi: 10.1186/s13071-016-1578-2 (PMC4890250; doi:10.1186/s13071-016-1578-2)
Supplement: Additional file 1: — 16S rRNA gene and COX1 sequences retrieved from GenBank. Each sequence is labelled as follows: Representative number associated to its corresponding Neighbour-net tree_Host_Location_Accession Number (DOC 85 kb) [file 13071_2016_1578_MOESM1_ESM.doc]

**Additional File 1.**

| 16S rRNA Sequences | COX1 Sequences |
| --- | --- |
| 1_Rabbit_Egypt_AB779582 | 1_Human_Australia_AY493385 |
| 2_Rabbit_Egypt_AB779583 | 2_Human_Australia_AY493388 |
| 3_Rabbit_Egypt_AB779580 | 3_Human_Australia_AY493390 |
| 4_Rabbit_Egypt_AB779579 | 4_Buffalo_Egypt_AB779589 |
| 5_Rabbit_Egypt_AB779578 | 5_Buffalo_Egypt_AB779590 |
| 6_Rabbit_Egypt_AB779576 | 6_Buffalo_Egypt_AB779591 |
| 7_Rabbit_Egypt_AB779575 | 7_Buffalo_Egypt_AB779593 |
| 8_Rabbit_Egypt_AB779574 | 8_Buffalo_Egypt_AB779592 |
| 9_Rabbit_Egypt_AB779573 | 9_Buffalo_Egypt_AB779595 |
| 10_Rabbit_Egypt_AB779572 | 10_Dog_Australia_AY493394 |
| 11_Buffalo_Egypt_AB779564 | 11_Dog_USA_AY493393 |
| 12_Cattle_Egypt_AB779581 | 12_Wallaby_Australia_AY493398 |
| 13_Sheep_Egypt_AB779586 | 13_Wombat_Australia_AY493397 |
| 14_Sheep_Egypt_AB779585 | 14_Dog_China_KJ499544 |
| 15_Sheep_Egypt_AB779584 | 15_Dog_China_KJ748528 |
| 16_Sheep_Egypt_AB779587 | 16_Dog_China_KJ748529 |
| 17_Rabbit_Egypt_AB779577 | 17_Dog_China_KJ748527 |
| 18_Buffalo_Egypt_AB779570 | 18_Dog_Australia_AY493391 |
| 19_Buffalo_Egypt_AB779571 | 19_Dog_Australia_AY493392 |
| 20_Buffalo_Egypt_AB779565 | 20_Dog_USA_AY493395 |
| 21_Buffalo_Egypt_AB779566 | 21_Human_Australia_AY493382 |
| 22_Buffalo_Egypt_AB779567 | 22_Human_Australia_AY493383 |
| 23_Buffalo_Egypt_AB779568 | 23_Human_Australia_AY493384 |
| 24_Buffalo_Egypt_AB779569 | 24_Chimp_Tanzania_AY493396 |
| 25_Dog_China_KJ781369 | 25_Buffalo_Egypt_AB779594 |
| 26_Dog_China_KJ781371 | 26_Cattle_Egypt_AB779607 |
| 27_Dog_China_KJ781370 | 27_Buffalo_Egypt_AB779588 |
| 28_Dog_China_KJ781373 | 28_Rabbit_Egypt_AB779604 |
| 29_Human_China_KJ781376 | 29_Rabbit_Egypt_AB779603 |
| 30_Human_China_KJ781378 | 30_Rabbit_Egypt_AB779605 |
| 31_Human_China_KJ781374 | 31_Rabbit_Egypt_AB779598 |
| 32_Human_China_KJ781377 | 32_Rabbit_Egypt_AB779597 |
| 33_Human_China_KJ781375 | 33_Rabbit_Egypt_AB779599 |
| 34_Dog_China_KJ781372 | 34_Rabbit_China_EU256389 |
| 35_Dog_Japan_AB821002 | 35_Swine_China_EU256387 |
| 36_Raccoon_Dog_Japan_AB820995 | 36_Rabbit_China_EU256388 |
| 37_Raccoon_Dog_Japan_AB821003 | 37_Rabbit_China_EU256386 |
| 38_Raccoon_Dog_Japan_AB820998 | 38_Sheep_Egypt_AB779608 |
| 39_Raccoon_Dog_Japan_AB820997 | 39_Sheep_Egypt_AB779609 |
| 40_Raccoon_Dog_Japan_AB820999 | 40_Sheep_Egypt_AB779602 |
| 41_Raccoon_Dog_Japan_AB820996 | 41_Sheep_Egypt_AB779610 |
| 42_Japanese_Marten_Japan_AB821000 | 42_Sheep_Egypt_AB779611 |
| 43_Japanese_Serow_Japan_AB821001 | 43_Rabbit_Egypt_AB779601 |
| 44_Dog_Australia_AY493410 | 44_Rabbit_Egypt_AB779606 |
| 45_Wallaby_Australia_AY493412 | 45_Rabbit_Egypt_AB779600 |
| 46_Chimp_Tanzania_AY493411 | 46_Rabbit_Egypt_AB779596 |
| 47_Human_Australia_AY493402 | 47_Human_China_KJ748524 |
| 48_Dog_Australia_AY493409 | 48_Human_China_KJ748523 |
| 49_Human_Australia_AY493403 | 49_Human_China_KJ748525 |
| 50_Human_Australia_AY493404 | 50_Human_China_KJ748521 |
| 51_Human_Australia_AY493408 | 51_Human_China_KJ748522 |
| 52_Human_Australia_AY493405 | 52_Human_China_KJ748526 |
| 53_Human_Australia_AY493406 | 53_Human_Panama_AY493379 |
| 54_Human_Australia_AY493407 | 54_Human_Panama_AY493380 |
| 55_Human_Panama_AY493401 | 55_Human_Panama_AY493381 |
| 56_Human_Panama_AY493399 |  |
| 57_Human_Panama_AY493400 |  |
| 58_Alpine_Chamois_Spain_AF311951 |  |
| 59_Red_Fox_23B_Italy_AF387691 |  |
| 60_Pyrenian_Chamois_Spain_AF387687 |  |
| 61_Red_Fox_23A_Italy_AF387695 |  |
| 62_Red_Fox_19D_Italy_AF387696 |  |
| 63_Alpine_Chamois_1A_Italy_AF387692 |  |
| 64_Alpine_Chamois_1B_Italy_AF387685 |  |
| 65_Alpine_Chamois_1C_Italy_AF387686 |  |
| 66_Red_Fox_19C_Italy_AF387697 |  |
| 67_Alpine_Chamois_18B_Italy_AF387698 |  |
| 68_Alpine_Chamois_5_Italy_AF387694 |  |
| 69_Alpine_Chamois_18A_Italy_AF387699 |  |
| 70_Alpine_Chamois_15H_Italy_AF387693 |  |
| 71_Alpine_Chamois_15G_Italy_AF387689 |  |
| 72_Alpine_Chamois_15F_Italy_AF387688 |  |
| 73_Red_Fox_20B_Spain_AF387700 |  |
| 74_Red_Fox_20C_Spain_AF387702 |  |
| 75_Red_Fox_20A_Spain_AF387701 |  |
| 76_Red_Fox_3A_Italy_AF387690 |  |
| 77_Red_Fox_4A_Italy_AF387680 |  |
| 78_Red_Fox_4B_Italy_AF387681 |  |
| 79_Red_Fox_16A_Italy_AF387676 |  |
| 80_Red_Fox_4C_Italy_AF387683 |  |
| 81_Red_Fox_6A_Italy_AF387679 |  |
| 82_Red_Fox_6B_Italy_AF387682 |  |
| 83_Red_Fox_8A_Italy_AF387675 |  |
| 84_Red_Fox_8C_Italy_AF387678 |  |
| 85_Red_Fox_8B_Italy_AF387677 |  |
| 86_Red_Fox_21_Italy_AF387684 |  |
| 87_Human_Italy_AF495527 |  |

16S rRNA and COX1 sequences retrieved from GenBank. Each sequence is labelled as follows: Representative number associated to its corresponding Neighbour-net tree_Host_Location_Accession Number.
